# Neoadjuvant chemotherapy alters the balance of effector to suppressor immune cells in advanced ovarian cancer

**DOI:** 10.1007/s00262-020-02670-0

**Published:** 2020-08-27

**Authors:** Alexandra Leary, Catherine Genestie, Félix Blanc-Durand, Sébastien Gouy, Ariane Dunant, Amandine Maulard, Françoise Drusch, Bianca Cheaib, Judith Michels, Enrica Bentivegna, Audrey LeFormal, Soizick Mesnage, Philippe Morice, Patricia Pautier, Aya S. Khairallah

**Affiliations:** 1 Gynecological Cancer Unit, Department of Medicine, Institut Gustave Roussy, Villejuif, France; 2 Pathology Department, Institut Gustave Roussy, Villejuif, France; 3 Biostatistics and Epidemiology Unit, Institut Gustave Roussy, Villejuif, France; 4 INSERM U981, Institut Gustave Roussy, Villejuif, France; 5 Department of Cancer Medicine, Breast Cancer Committee, Institut Gustave Roussy, Villejuif, France; 6 Medical Oncologist, Gynecology Unit, INSERM U981, Institut Gustave Roussy, 114 rue Edouard Vaillant, 94805 Villejuif, France

**Keywords:** Ovarian cancer, Microenvironment, TILs, Neoadjuvant chemotherapy, Immunotherapy

## Abstract

**Background:**

At diagnosis, tumor-infiltrating lymphocytes (TILs) are prognostic in epithelial ovarian cancer (EOC). We recently demonstrated that neoadjuvant chemotherapy (NACT) significantly increased stromal TILs. Here, we investigated the impact of NACT on immune subpopulations with a particular focus on the balance of immune-reactive to tolerant subpopulations.

**Materials and methods:**

Tissue microarrays of EOC (145 pre-NACT, 139 post-NACT) were analyzed for CD3+, CD8+, FOXP3+, CD68+, and CD163+ by immunohistochemistry and CD4+ cells from deduction. Stromal TILs scored as percentage of stromal area, while intra-epithelial TILs scored as number of TILs in contact with tumor cells/HPF. Differences were evaluated by Wilcoxon or Chi square tests, Wilcoxon signed-rank for paired analyses, and cox model for PFS and OS.

**Results:**

NACT significantly increased stromal CD3+ (*p* = 0.003) and CD8+ (*p* = 0.001) and intra-epithelial CD8+ (*p* = 0.022) and CD68+ (*p* = 0.0003) infiltration in unmatched samples and among paired samples for stromal CD3+ and CD8+. Neither CD3+, CD8+, CD4+, and CD68+ nor CD163+ expression correlated with outcome at diagnosis or post NACT. Using median value as a cut-off, high stromal CD8+/FOXP3+ ratio (HR = 0.59; *p* = 0.017) and high stromal CD3+/FOXP3+ ratio post NACT were associated with prolonged PFS (*p* = 0.0226). The more the balance shifted in favor of effector versus regulatory TILs, the better the survival. Similarly, high CD68+/CD163+ ratio post NACT improved PFS (*p* = 0.0445).

**Conclusion:**

NACT has a significant impact on the balance of immune-reactive to immune-tolerant subpopulations and a high ratio of CD8+/FOXP3+, CD3+/FOXP3+, and CD68+/CD163+ post NACT was significantly associated with improved outcomes. Whether this could select patients for immunotherapy in the post-operative setting should be investigated.

**Electronic supplementary material:**

The online version of this article (10.1007/s00262-020-02670-0) contains supplementary material, which is available to authorized users.

## Background

The majority of patients with epithelial ovarian cancer (EOC) present with advanced tumor stage (FIGOIII/IV) propelling it to the most lethal gynecologic malignancy. Indeed, EOC affects approximately 1 in 70 women with only 45% surviving 5 years after diagnosis [1]. The backbone of management rests on complete resection of all macroscopic disease and platinum-based chemotherapy [2]. In advanced disease where complete resection cannot be achieved, neoadjuvant chemotherapy (NACT) followed by interval debulking surgery and adjuvant chemotherapy is a suitable alternative [3, 4].

There is increasing evidence suggesting that measures of local anti-tumor immunity may be relevant prognostic factors in several neoplasms including ovarian cancer. Tumor-infiltrating lymphocytes (TILs) have been repeatedly associated with improved survival in EOC [5–8]. Infiltration of CD3+ T-cells, and particularly CD8+ cytotoxic cells, is most strongly and consistently associated with a better prognosis in many independent studies [5, 7, 9–14].

On the other hand, immune-suppressive cells, such as FOXP3+ regulatory T-cells (Tregs), also present in the tumor bed, are often associated with a negative prognostic impact [5, 15]. Similarly, tumor-associated macrophages (TAMs) have been shown to predict an unfavorable prognosis, in particular, the M2-polarized (CD163+) subset [5, 16]. In fact, the interaction between the immune system and the tumor is likely based on an equilibrium between immune recognition and immune tolerance so that a measure of the balance between immune activating and immune-suppressive cells may be more informative. Accordingly, studies have reported that high ratios of intra-tumoral CD8/Tregs or CD68/CD163 macrophages were associated with improved overall survival in ovarian cancer [5, 16, 17].

Unfortunately, most studies have focused on chemotherapy naïve tumors. Emerging data suggest that conventional cytotoxics may alter the local immune state by inducing adaptive stress response in malignant cells, hence operating as immunological adjuvant, or by depleting tumor-infiltrating immunosuppressive cells [18–20].

The neoadjuvant setting where paired ovarian tumor samples are obtained at diagnosis and at interval debulking after platinum-based chemotherapy offers a useful model to study the impact of cytotoxic treatment on anti-tumor immunity. We have recently shown that neoadjuvant chemotherapy (NACT) had a significant impact on the ovarian tumor immune microenvironment by increasing TIL infiltration and PDL1 expression [21]. It is, therefore, crucial to define which immune subsets are actually recruited to the tumor bed post NACT, and specifically whether these are involved in immune surveillance or immune tolerance.

Our aim was to further elucidate the impact of chemotherapy on the tumor immune landscape by characterizing the changes in subsets of T-cells and macrophages with NACT in a large cohort of clinically annotated ovarian cancer samples before and after NACT with a particular focus on the balance of putative immune-reactive to immune-tolerant subpopulations. Immune parameters before and after NACT were also related to clinical outcome to evaluate their prognostic value.

## Materials and methods

### Tissue microarray (TMA)

A clinically annotated TMA of epithelial ovarian cancer (EOC) samples from the Gustave Roussy Cancer Center biobank was constructed as previously published from 150 patients providing 133 pre-chemotherapy samples obtained at diagnostic laparoscopy or primary surgery and 128 post-chemotherapy samples obtained at interval surgery. Samples were selected based on the greatest viable tumor cellularity; necrotic samples were excluded. In few patients with complete pathological response, immune parameters were not evaluated. The presence of tumor in the arrayed samples was confirmed by H&E staining.

### Immunohistochemistry

Immunohistochemical staining of TMA slides of formalin- or acetic acid formalin alcohol (AFA)-fixed samples was performed by the autostainer Benchmark Ultra (ROCHE). The slides were dewaxed and rehydrated. Antigen retrieval was performed using a citrate-based antigen unmasking solution (CC1) for 36 min. The following antibodies were used with UltraView Kit: rabbit anti-human CD3 antibody (DAKO; clone:polyclonal) (1/100) for1 h at 37 °C, rabbit anti-human CD8 antibody (Spring Bioscience; clone: SP16) (1/100) for 1 h at 37 °C, rabbit anti-human FOXP3 antibody (Spring Bioscience; clone: SP97) (1/100) for 1 h at 37 °C with ampli, mouse anti-human CD68 antibody (DAKO; clone:KP1) (1/1000) for 20 min at 37 °C, and mouse anti-human CD163 antibody (DPS, Cliniscience; clone:10D6) (1/75) for 44 min at room temperature.

### Histopathological analysis and TILs assessment

Immunostained TMAs were examined and scored by two pathologists (A.K. and C.G.) blinded to clinical data. For every marker, a mean score from three cores from each sample was given after evaluation at 20 × and 40 × objective lens. The assessment of marked TILs was done following the guidelines described by the international TILs Working Group 2014 [22] : The boundaries of the invasive tumor were identified and only TILs inside them were evaluated (Supplementary Fig. 1a). Stromal TILs (sTILs) were defined as lymphocytes located in the tumor stroma between the carcinoma cells, but with no direct contact with them, while intra-epithelial TILs (ieTILs) were defined as lymphocytes in tumor nests having cell-to-cell contact with no intervening stroma and in direct interaction with carcinoma cells (Supplementary Fig. 1b). Since both types of TILs are localized in the region defined as tumor tissue, both categories represent true TILs.

Stromal subsets of TILs were scored as the percentage of total intra-tumoral stromal area occupied by CD3+, CD8+, or FOXP3+ mononuclear cells. Since no international recommendations are available for intra-epithelial TILs (ieTILs) scoring and methodologies vary widely in previous reports, they were quantified by counting the average of number of intra-epithelial TILs per high-power field(× 40) on 5 fields. CD4+ stromal and ieTILs were estimated by subtracting CD8+ cells from CD3+ cells.

Since TMA spots were lost with each additional section, both macrophage markers (CD68 in red and CD163 in brown) were performed on the same slide by double labeling and were determined as the percentage of positive cells within the tumor area. Because of the double-staining, the boundaries between stroma, macrophages, and epithelial cells were often unclear, so CD68 and CD163 scoring encompassed both stromal and intra-epithelial compartments.

TILs in areas with crush artifacts, necrosis, or extensive regressive hyalinization were not scored. A necrotic biopsy was considered not assessable.

### Statistical analysis

All statistical analyses were performed using SAS 9.3. The cut-off to define high/low for each marker was the median value rounded to the nearest integer. Continuous variables were compared with Wilcoxon tests. Comparisons of categorical variables were performed using Chi square tests which were exact when appropriate. For paired samples, the Wilcoxon rank sign test was used, after checking that for most differences of marker expressions, the normality assumption was violated.

Progression-free survival (PFS) was calculated from the date of histological diagnosis to progression or death. Survival curves were generated using the Kaplan–Meier Method and analyses were performed with a Cox model.

## Results

### Clinical patient profile

The clinical and histological characteristics of patients included in this study were previously published (Supplementary Table 1) [21]. In brief, patients with newly diagnosed Stage III/IV epithelial ovarian cancer were treated with platinum and paclitaxel neoadjuvant chemotherapy (mean number of cycles = 4) with the goal of achieving complete cytoreduction. The median duration of follow-up was 80 months (95% CI 68–93 months), median PFS for the whole cohort was 20 months (95% CI 18–23 months), and median overall survival (OS) was 45 months (95% CI 39–62 months).

### Changes in T-cell subsets with NACT

The number of samples evaluable for each marker varied due to crush artifacts, or because samples were lost as the TMA was increasingly sectioned.

Total CD3+ T-cells, as well as cytotoxic CD8+, helper CD4+, and regulatory FOXP3+ T-cells were evaluated in the stromal and intra-epithelial compartments in tumor samples before and after NACT.

At diagnosis, stromal CD3+ TILs were detected in all tumor samples but showed wide variability among tumor samples (*N* = 110, median: 15%; range 1–57%). This inter-patient heterogeneity was also observed for stromal CD8+ (*N* = 107, median: 10%; range 0–40%), CD4+ (*N* = 106, median: 5%; range 0–32%), and FOXP3+ TILs (*N* = 86, median: 5%; range 0–25%). In contrast, intra-epithelial TILs were found in fewer cases. Median for intra-epithelial CD3+ (range 0–30 cells/HPF) was 2 cells/HPF, and only 1 cell/ HPF for CD8+ (range 0–18 cells/HPF), CD4+ (range 0–19 cells/HPF), and FOXP3+ TILs (range 0–7 cells/HPF).

**Fig. 1:**
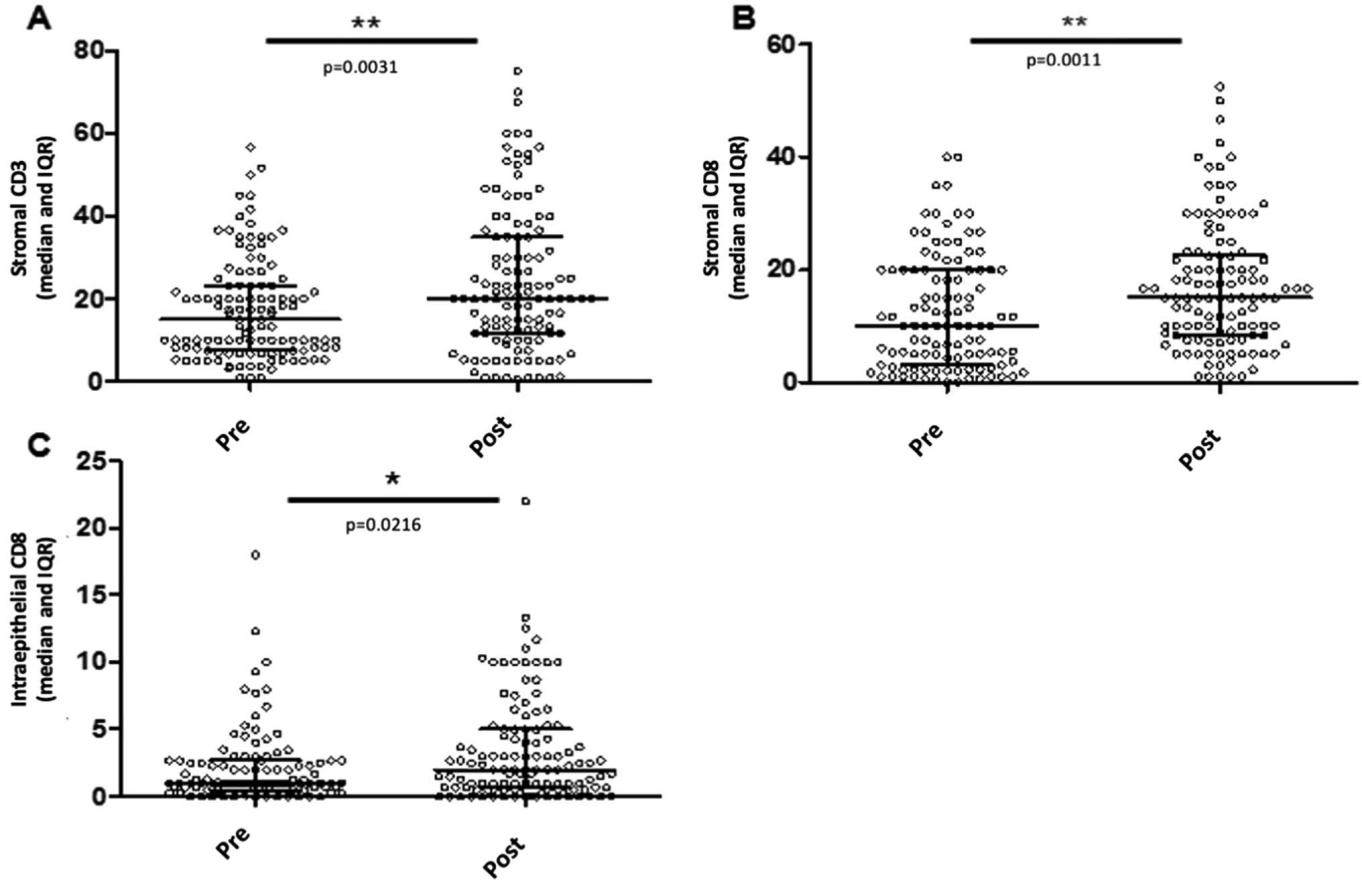
Comparison of immune cell infiltrates in specimens of EOC patients prior and after neoadjuvant chemotherapy. **a** Changes in stromal CD3*+* TILs (%); **b**, **c** changes in stromal (%) and intra-epithelial (/HPF) CD8*+* TILs

Overall, stromal CD3+ T-cells increased significantly after NACT (median: 15% to 20% pre to post; *p* = 0.003, Fig. 1a). Similar increases were noted in stromal CD8+ TILs (*p* = 0.001, Fig. 1b) and in CD8+ ieTILs (*p* = 0.022, Fig. 1c). There was no significant difference in median CD4 or FOXP3 stromal/intra-epithelial scores before and after NACT (data not shown).

**Fig. 2:**
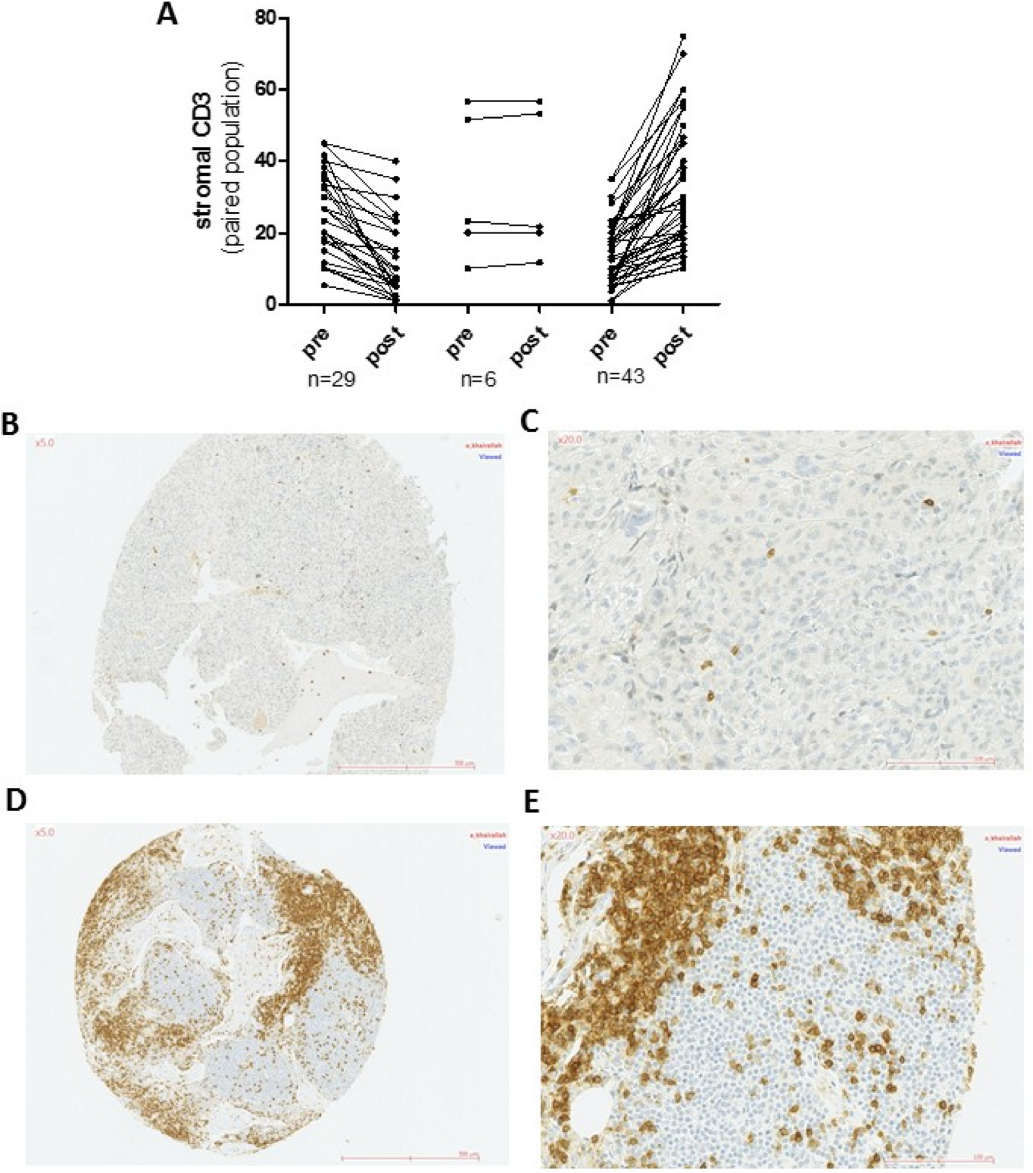
**a** changes in CD3 expression in immune cells in paired pre- and post-NACT tumors, in the stromal compartment (%). CD3 expression in paired tumors from a single patient showing an increase in CD3 expression after therapy. Pre-NACT, B(×5), C(×20) and post-NACT D(×5), E(×20)

**Fig. 3:**
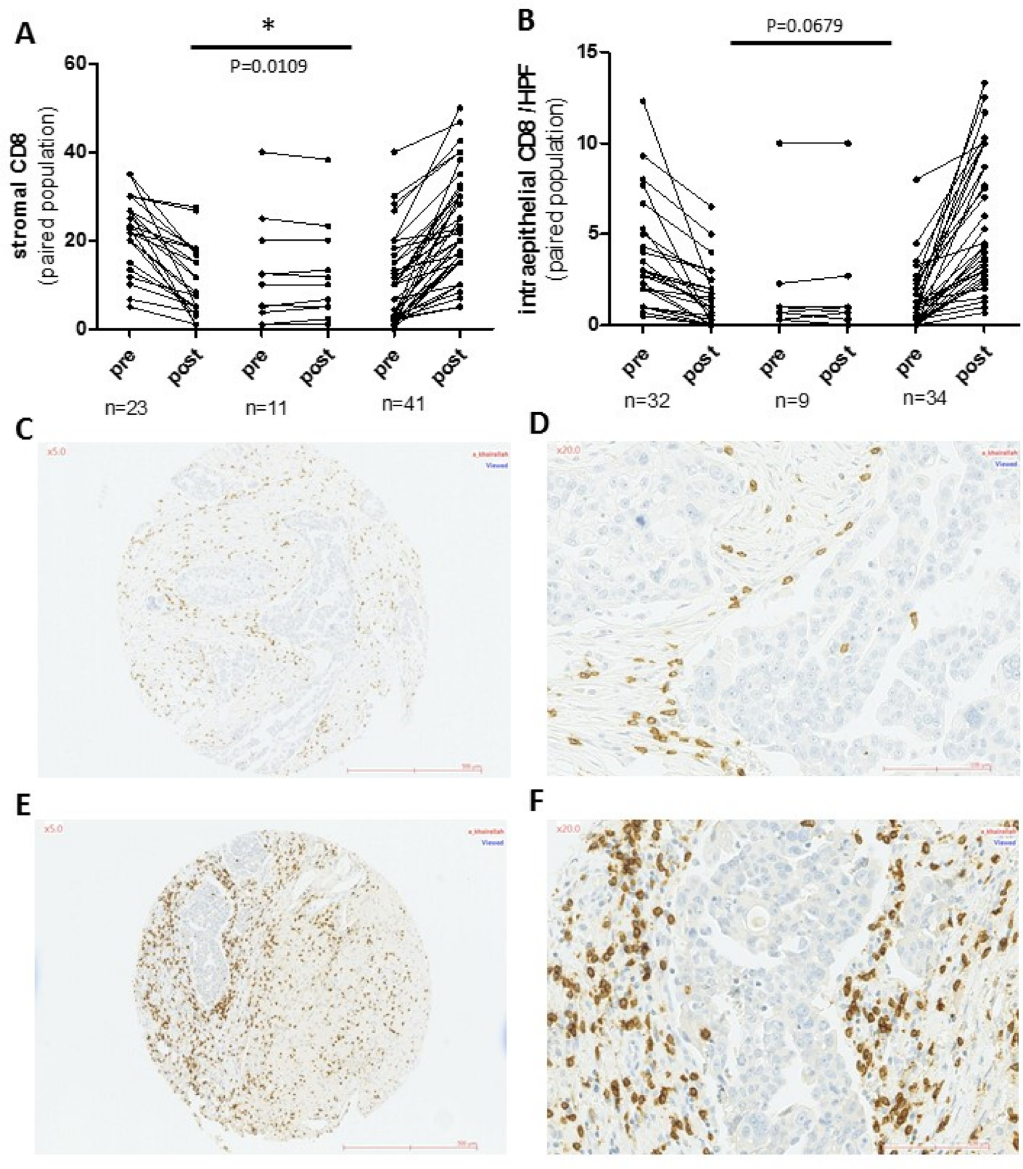
Changes in CD8 expression in immune cells in paired pre- and post-NACT tumors; **a** in the stromal compartment (%) and **b** in the intra-epithelial compartment (/HPF). CD8 expression in paired tumors from a single patient showing an increase of CD8 expression after therapy. Pre-NACT, C(×5), D(×20) and post-NACT E(×5), F(×20)

Among the smaller subset of patients with matched paired pre- and post-NACT samples, a significant increase in median stromal CD3+ (*p* = 0.02, *N* = 77) and stromal CD8+ (*p* = 0.008, *N* = 73) was also observed. However, the impact of NACT was clearly variable among individual patients with 55% of the patients (43/77) showing increased, 37% (29/77) showing decreased, and 8% (6/77) showing unchanged stromal CD3+ TILs post NACT (Fig. 2a–e). Similar patterns were observed with stromal CD8+ TILs (Fig. 3a–f) highlighting the inter-tumor variability in immune responses to NACT.

**Fig. 4:**
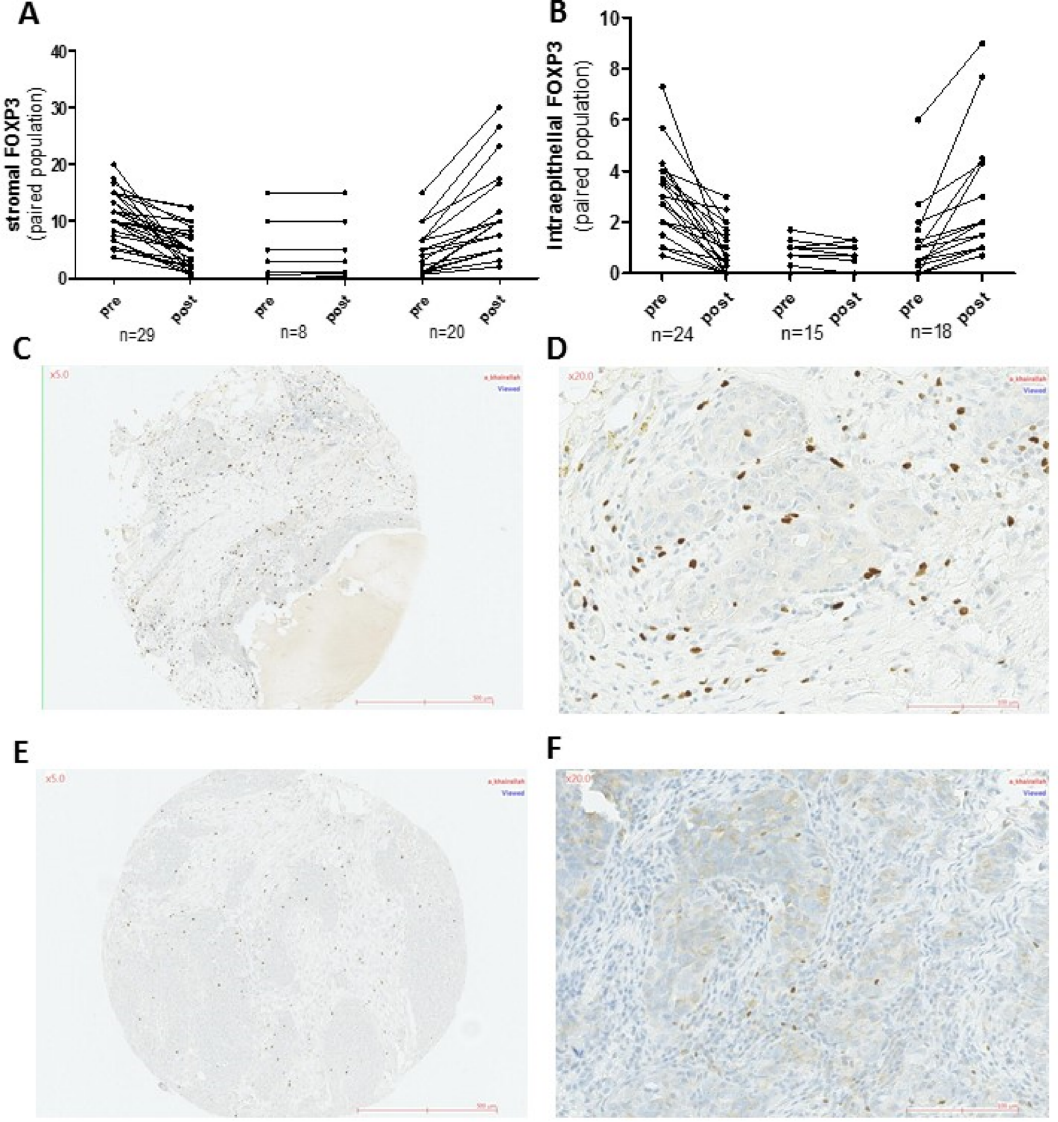
Changes in FOXP3 expression in immune cells in paired pre- and post-NACT tumors; **a** in the stromal compartment (%) and **b** in the intra-epithelial compartment (/HPF). FOXP3 expression in paired tumors from a single patient showing a decrease in FOXP3 expression after therapy. Pre-NACT, C(×5), D(×20) and post-NACT E(×5), F(×20)

With regards to FOXP3+ TIL infiltration, there was no overall difference in the median stromal or intra-epithelial scores; however, when considering paired samples from individual patients, the pattern of change in FOXP3+ infiltration varied markedly among patients. Half the patients (27/54) showed decreasing FOXP3+ stromal infiltration, while 38% demonstrated stromal FOXP3+ + TIL recruitment post NACT. With regards to ieFOXP3+ TILs, 33% exhibited increasing infiltration, while 50% of patients showed diminishing levels, including 15% becoming completely ieFOXP3-negative post NACT (Fig. 4a–f).

Given the heterogeneous patterns of change in TIL subpopulations and the fact that the balance of immune-reactive versus immune-tolerant cells may be more relevant to deciphering the tumor immune contexture, the ratios of CD8+/FOXP3+, CD4+/FOXP3+, and CD3+/FOXP3+ in individual tumors were derived. Overall, the median of CD8+/FOXP3+ ratios increased significantly post NACT in the stromal (*p* = < 0.0001) and intra-epithelial compartments (*p* = 0.0007) as well as the median of intra-epithelial CD3+/FOXP3+ ratios (*p* = 0.006) favoring anti-tumor immunity. Also, the proportion of tumors demonstrating a favorable ratio (e.g., ratio of effector to suppressor TILs > 1) increased post NACT from 71% to 95% (Chi square < 0.0001) and from 48% to 78% (Chi square < 0.0001) for stromal and ieCD8+/FOXP3, respectively (Table 1). With regards to CD4+/FOXP3+ ratios, the proportion of tumors showing an intra-epithelial CD4+/FOXP3+ ratio > 1 increased from 42% to 60% (Chi square = 0.002) with NACT, again suggesting that NACT shifted the balance towards effector versus suppressor T-cells. Stromal CD4+/FOXP3+ ratio did not change significantly post NACT (Table 1).

**Table 1:** Proportion of tumors showing a favorable effector to suppressor ratio (e.g., ratio > 1) pre-NACT and post-NACT

Ratios	Pre-NACT		Post-NACT		*P* value (chi square)
	*N*	% tumors with ratio > 1	*N*	% tumors with ratio > 1	
Stromal CD8/FOXP3					
> 1	60	71%	95	95%	< 0.0001
≤ 1	24		5		
ieCD8/FOXP3					
> 1	34	48%	60	78%	< 0.0001
≤ 1	37		17		
Stromal CD4/FOXP3					
> 1	46	54%	51	49%	NS
≤ 1	39		53		
ieCD4/FOXP3					
> 1	30	42%	48	60%	0.002
≤ 1	41		32		
CD68/CD163					
> 1	8	14%	24	35%	0.0003
≤ 1	52		45		

### Changes in CD68+ and CD163+ macrophages with NACT

Subsets of TAMs were evaluated using CD68, a non-specific macrophage marker, and CD163, a putative marker for M2-polarized pro-tumoral macrophages. At diagnosis, tumors showed a greater prevalence of CD163+ than CD68+ TAMs. The median CD68+ score was 2.5 (*N* = 71, range 0–30%) compared to a median CD163+ score of 10 (*N* = 74, range 0–45%) suggesting that in treatment naïve EOC the macrophage compartment is primarily driven by pro-tumoral M2-like cells.

Post NACT, infiltration by CD68+ TAMs significantly increased to a median of 10 (*N* = 73, *p* = 0.0003, Supplementary Fig. 2a), while no significant overall change in median CD163+ TAMs was noted (median: 10, *N* = 72, *p* = NS, Supplementary Fig. 2b). But as for FOXP3+, when considering paired samples from individual patients, the impact of NACT on CD163+ infiltration was variable with 42% showing an increase in CD163+ cells post NACT, 46% showing a decrease, and 12% showing stable levels (Supplementary Fig. 2d). As a result, when considering the ratio of CD68+/CD163+ TAMs in individual tumors, there was a significant change in the balance of CD68+ to CD163+ macrophages with NACT. At diagnosis, the macrophage infiltration was dominated by CD163+ cells; however, after NACT the proportion of tumors showing a greater infiltration of CD68 than CD163 macrophages increased significantly by over twofold from 14% to 35% (Chisquare-*p* = 0.0003, Table 1).

### Correlation of TIL and TAM subsets with clinical outcome

When considering standard prognostic factors (age, stage, BRCA status and completeness of resection), only completeness of resection (CC0 versus other) was significantly associated with PFS (HR = 0.31; *p* < 0.0001). Contrary to the literature showing that density of CD3+ and CD8+ TILs pre-treatment is strongly associated with survival [5, 14], in our study focusing on patients with unresectable OC treated with NACT, none of the T-cell or macrophage subsets, in either stromal or intra-epithelial compartment, correlated to PFS in pre-NACT samples. The prognostic impact of levels of immune infiltration post NACT was examined next. High stromal FOXP3 was the only immune marker associated with PFS (HR = 1.7; *p* = 0.03), but not OS. Neither CD3+, CD8+, CD4+ TILs, and CD68+ TAM nor CD163+ TAM showed any significant correlation with PFS or OS after neoadjuvant chemotherapy.

### Integrating the balance of immune-reactive to immune-tolerant subpopulations for prognostic information

**Fig. 5:**
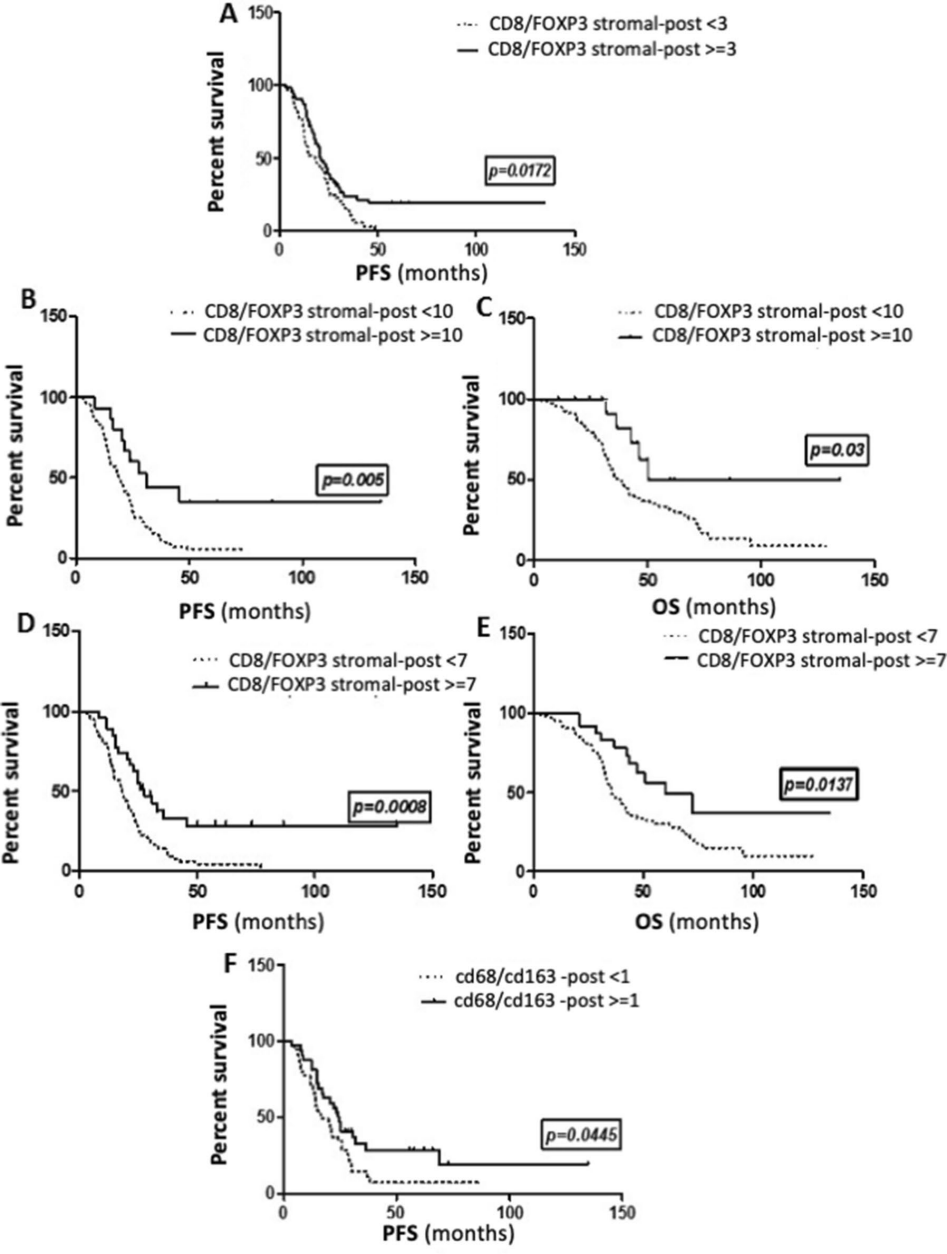
Kaplan–Meier curves showing the correlation of the ratios of effector to tolerant immune infiltrates with survival in ovarian cancer. Post-NACT stromal CD8/FOXP3 ratio and PFS, with the median as a cut-off (**a**); with a cut-off of 10 (**b**); and OS with a cut-off of 10 (**c**). Post-NACT stromal CD3/FOXP3 ratio and PFS (**d**) and OS (**e**) with a cut-off of 7. Post-NACT CD68/CD163 ratio and PFS with median as a cut-off (**f**)

Given recent reports that the balance between effector and suppressor immune subsets may offer more useful prognostic information [5, 23–25], we evaluated the correlation between ratios of CD8+/FOXP3+, CD3+/FOXP3+, CD4+/FOXP3+, and CD68+/CD163+ and survival in tumor samples at diagnosis and after NACT. None of the ratios were prognostic in pre-treated samples. However, using the median value as a cutoff (≥ 3), a high stromal CD8+/FOXP3+ ratio post NACT was associated with a significant improvement in PFS (HR = 0.57; *p* = 0.009, Fig. 5a). In fact, the more the balance in the stromal tumor microenvironment post NACT shifted in favor of CD8+ over FOXP3+ TILs, the better the survival (test for trend, *p* = 0.01), such that with a cutoff of ≥ 10, a high stromal CD8+/FOXP3 post NACT was associated with a significant improvement in both PFS (HR =  0.48; median: 19 vs. 31mo, *p* = 0.005, Fig. 5b) and OS (HR = 0.49; median: 37 vs. 51mo, *p* = 0.03, Fig. 5c). Post NACT CD8+/FOXP3+ ieTIL cell ratio was not correlated with clinical outcome.

Similarly, PFS was significantly increased in tumors with a high stromal CD3+/FOXP3+ ratio post NACT (HR = 0.6, *p* = 0.02). High stromal CD4+/FOXP3 also significantly predicted improved PFS (HR = 0.64; *p* = 0.04) and OS (HR = 0.46; *p* = 0.003); again, the higher the ratio, the greater the impact on OS (*p* = 0.01). High CD68+/CD163+ ratio showed a trend for improved PFS and OS, but neither were significant.

On multivariate analysis, after adjusting for completeness of surgery, the ratio of stromal CD8+/FOXP3 post NACT remained significantly associated with PFS (HR = 0.62; *p* = 0.03), while the ratio of stromal CD4+/FOXP3+ post NACT was significantly predictive of OS (HR = 0.46; *p* = 0.003).

## Discussion

We have previously reported that NACT had a marked impact on the tumor immune microenvironment, resulting in significant increases in TIL infiltration and PDL1 expression [21]. In the current study, we sought to characterize which immune subpopulations were actually recruited to the tumor bed, including T-cells and macrophages with a particular focus on the balance of effector to suppressor cells.

We have demonstrated that NACT significantly altered the balance of immune effector to suppressor T-cells and macrophages in favor of anti-tumor immunity. Overall, NACT was associated with significant increases in anti-tumor CD8+, CD3+ TILs, and CD68+ macrophages and had a more variable impact on suppressive FOXP3+ TILs and CD163+ macrophages. When considering ratios of effector to suppressor immune subsets in individual patients, we observed a significant increase in the proportion of tumors showing favorable ratios of CD8+/FOXP3+ stromal and ieTILs (*p* < 0.0001), CD4+/FOXP3+ ieTILs (p = 0.002), and CD68+/CD163+ macrophages (*p* = 0.0003) post NACT. Importantly, high CD8+/FOXP3+, CD4+/FOXP3+, and CD3+/FOXP3+ post NACT were significantly predictive of both improved PFS and OS, while high CD68+/CD163+ post NACT was non-significantly associated with improved PFS. In contrast, baseline ratios were not prognostic. Taken together, these data suggest that the balance of immune-tolerant to reactive cells in the residual tumor post NACT may be a significant driver of increased survival and that the prognosis of women with advanced EOC may be further improved by harnessing anti-tumor immunity during or after primary treatment.

We were unable to analyze the correlation between these ratio and response rates without risking significant bias. Indeed, response to chemotherapy is notoriously difficult to objectively measure in EOC. Peritoneal disease is poorly visualized on CT scans, Ca125 diminishes in 90% of patients, and scores such as the sugarbaker score are limited to frequent incomplete evaluation of the abdomen during diagnostic laparoscopy resulting in truncated scores. The chemotherapy response score (CRS) is an interesting option, and we attempted to perform it retrospectively; unfortunately, the block selected for the TMA construction was the one containing the most viable tumor tissue, frequently an omental biopsy, and is also the block that is recommended for the CRS.

Recent evidence suggests that antineoplastic agents may exert therapeutic effects by increasing the host’s immune anti-tumor response via a host of mechanisms. Anthracyclines or oxaliplatin have been shown to induce immunogenic cell death (ICD), thereby activating anti-tumor immunity [26]. With regard to cytotoxics conventionally used in ovarian cancer, neoadjuvant cisplatin increased the intra-tumoral trafficking of CD4 and CD8 T-cells in both mice models and patients with esophageal cancer [27], while paclitaxel augmented cellular immunity in patients with advanced non-small cell lung cancer by increasing circulating CD8 T-cells and IL-2-secreting CD4 T-cells [28]. Taxanes have also been reported to specifically impair the viability of FOXP3^+^ Treg cells [29] or to polarize macrophages toward an M1-like phenotype and to selectively kill myeloid-derived suppressor cells while sparing the M1-like macrophages [30]. Taken together, these data support that neoadjuvant platinum- and taxane-based treatment may indeed modulate the tumor immune microenvironment.

We showed that NACT resulted in significant increases in total and cytotoxic T-cells. These results are in agreement with prior studies in EOC [8, 23, 31, 32]. Our study showed a significant increase in CD68+ TAMs after chemotherapy. In line with our findings, DeNardo et al reported that cytotoxics, such as cisplatin or paclitaxel, favored recruitment of macrophages to neoplastic lesions and potentiated their cytotoxicity [33, 34].

Despite significant increases in CD3+, CD8+, and CD68+ cells post NACT, no immune subsets on their own were prognostic. This is in line with previous studies also failing to demonstrate a significant correlation between increased CD8+ or CD68+ cells post NACT and survival [8, 23, 31, 32].

Studies have demonstrated that accumulation of FOXP3+ regulatory T-cells in EOC limits anti-tumor immunity and favors tumor cell growth, thereby reducing patient survival.

In the current study, there was no significant change in median FOXP3+ levels. However, medians in the whole group fail to accurately capture the heterogeneous immune responses to NACT. When looking at paired samples from individual patients, half showed decreasing FOXP3+ TILs and FOXP3+ infiltration post NACT in individual patients was significantly correlated with outcome: patients with low FOXP3+ TILs post NACT had significantly prolonged PFS (HR = 0.520; *p* = 0.005). The same inter-patient variability in immune response to NACT was observed across all TIL and macrophage subsets (Fig. 1) and likely reflects heterogeneity in host and tumor factors in patients with advanced EOC. These results underscore the critical importance of evaluating the post-NACT tumor in individual patients in order to integrate data on the diagnostic tumor immune profile and its evolution with chemotherapy [21].

The balance between anti-tumor versus pro-tumor immune cells in individual patients post NACT was most significantly correlated with outcome. Increasing data suggest that cytotoxics can have a concerted effect both recruiting and activating immune effectors while inhibiting immune-tolerant subpopulations [34].

In the current study, high CD8+/FOXP3+ T-cell ratio after NACT was associated with significantly better survival. Using the median as a cut-off, high CD8+/FOXP3+ post NACT predicts improved PFS (HR = 0.59; *p* = 0.017). Importantly, the positive prognostic value of CD8+/FOXP3+ increases as the ratio increases, so that using a cut-off of > 10, high CD8+/FOXP3+ is associated with a significant 12 months improvement in PFS (HR =  0.48; 19 vs 31 months, *p* = 0.005) and a significant increase in OS (HR = 0.49; median: 37 vs. 51 months, *p* = 0.03). After NACT, it is the combination of high CD8+ TILs which recognize foreign antigens on tumor cells and kill it by inducing the release of perforin and granzyme, associated with decreased FOXP3+ TILs, which have a critical role in suppressing anti-tumor immunity, leading to improved survival. And the greater the difference between these two subsets, the more the outcome is improved. Similar observations were made in breast cancer patients treated with NACT where a high CD8+/FOXP3+ ratio in residual tumors was prognostic [35]. Granzyme B is a granule-associated protein crucial for cytolytic function constitutively expressed by NK cells and activated CD8+ cells. Polcher et al. [23] showed a strong correlation between high granzyme B+/FOXP3+ ratio post NACT and improved PFS among 30 paired EOC samples. Together, these data support the prognostic relevance of the balance between immune activator and suppressor T-cells recruited to the tumor bed after NACT.

Similarly, the differential recruitment of subsets of macrophages to the tumor microenvironment provided the most prognostic information. Overall, CD68+ macrophages increased significantly post NACT, while median CD163+ remained stable; however, the pattern of change in CD163+ showed significant inter-patient variability. Macrophages can be classically (M1) or alternatively (M2) activated. M1 macrophages produce pro-inflammatory cytokines such as IL-12 and IL-23 or nitric oxide and demonstrate antigen-presenting activity, thus promoting tumor cell killing. Robust IHC markers for M1 markers are lacking; however, CD68 is a non-specific macrophage marker. In contrast, M2 macrophages which release immunosuppressive cytokines/chemokines are involved in immune tolerance and promote tumor growth and metastasis. The CD163 antigen is strictly expressed in monocytes/macrophages and is one of the markers used to identify M2 macrophages [36]. High levels of CD163+ macrophages in EOC samples at diagnosis have been shown to predict poor prognosis [37]. However, there is increasing data to suggest that it is in fact the balance between M1 and M2 subsets that is the most relevant. A high CD163+/CD68+ ratio at diagnosis has been shown to be a stronger predictor of OS, than CD163+ alone [37] and Zhang et al. demonstrated that a high M1/M2 ratio was associated with extended survival in ovarian cancer, whereas neither subtype alone was prognostic [38]. In the current study, CD163+ macrophages post NACT on their own did not predict outcome probably due to the tremendous overall increase in CD68+ cells, including M1 macrophages, which may counteract the immune-suppressive activity of CD163+ cells. Again, it was the balance of immune-reactive versus tolerant macrophages post NACT that was most informative. Patients with tumors showing a high CD68+/CD163+ ratio post NACT had a slight improvement in PFS (16.5 vs 24 months, *p* = 0.044), but lacked significance due to small numbers.

Despite recent meta-analyses providing evidence that high intra-epithelial CD3+, CD8+, or CD103+ TILs [39] and high CD68+/CD163+ ratios in EOC samples at diagnosis are predictive of survival [16], we could find no association between immune parameters at diagnosis and PFS or OS. Consistent with our findings, no association of pre-NACT CD3+, CD8+, FOXP3+, CD20+, or PDL1+ TILs with prognosis has been documented in several other studies [23, 31, 32]. Some aspects contributing to these conflicting results may include the method of assessing TILs, the use of different antibodies, as well as disease stage, immune status, and treatment pathways of the patients included. Indeed, ours and other studies showing no association of immune subsets at diagnosis with prognosis included only patients with bulky disease treated with NACT, while patients included in the meta-analyses were largely treated with upfront surgery. Advanced disease or neoadjuvant treatment may influence the prognostic value of baseline immune parameters, making those of post NACT more relevant.

In this report, stromal TILs offered the most prognostic information. Previous studies have largely described correlations between intra-epithelial TILs and survival in EOC. However, there is currently no standardized approach to evaluate TILs in EOC; the difference between intra-epithelial/stromal/intra-tumoral/peritumoral TILs is poorly defined and often mingled. In contrast, in breast cancer, a recommended method of evaluation has been published by the international TILs Working Group where both stromal TILs and intra-epithelial TILs have been well defined and assessed as independent parameters, both representing true intra-tumoral TILs. Based on recent studies on breast cancer, stromal TILs have emerged as a stronger, more reproducible prognostic marker than intra-epithelial TILs [22, 40–42]. We had chosen to use their recommended definitions and method of evaluation in our study to differentiate intra-epithelial from stromal TILs and to evaluate stromal TILs.

In summary, the current study is, to our knowledge, the biggest study describing the impact of carboplatin/taxane-based NACT on the lymphocyte and macrophage composition of advanced epithelial ovarian cancer. In fact, we add more power to previous studies by showing that neoadjuvant chemotherapy has the capacity to alter the influx and phenotype of immune infiltrates by significantly increasing overall CD3+, CD8+ cytotoxic cells, and CD68+ TAMs and decreasing FOXP3+ regulatory TILs or CD163+ macrophages in nearly half the patients. Importantly, relative infiltration by immune subtypes post NACT was prognostic. Using the median as a cut-off, high CD8+/FOXP3+, high CD3+/FOXP3+, and high CD68+/CD163+ in treated samples were all significantly predictive of improved PFS. In addition, the more the ratio shifted in favor of effector versus regulatory T-cells, the greater the prognostic benefit such that patients with CD8+/FOXP3+ or CD3+/FOXP3+ ratios greater than 10 and 7, respectively, had significant improvements in both PFS and OS. These data support that in a subset of patients, NACT may modulate the equilibrium of immune subsets in favor of immune rejection, thus contributing to tumor eradication and improved outcomes. Moreover, the impact of NACT on the tumor immune microenvironment showed significant inter-patient variability. This likely reflects heterogeneity in tumor and host factors in advanced EOC and could have important implications for personalized immunotherapy. We recommend further studies on bigger cohorts to better elucidate the immune changes occurring at the tumor bed after NACT. In the future, an individualized approach based on the type of immune subpopulations and expression of co-regulatory molecules identified in tumor at interval debulking after NACT could be proposed and may represent a promising approach to harness anti-tumor immunity, eradicate minimal residual disease, and result in meaningful improvements in outcome for patients with advanced ovarian cancer.

### Electronic supplementary material

Below is the link to the electronic supplementary material.

Supplementary file1 (DOCX 145 kb)

## Data Availability

Datasets generated for the current study are not publicly available considering confidentiality reasons. Anonymized data may be available from the corresponding author on justified request.
