# Supplementary material for: Neoadjuvant chemotherapy alters the balance of effector to suppressor immune cells in advanced ovarian cancer
Source: Cancer Immunol Immunother. 2020 Aug 27;70(2):519–31. doi: 10.1007/s00262-020-02670-0 (PMC7889679; doi:10.1007/s00262-020-02670-0)
Supplement: Supplementary file 1 — Supplementary file1 (DOCX 145 kb) [file 262_2020_2670_MOESM1_ESM.docx]

**APPENDIX A**

Supplementary Tables :

|  | **No. pts** | **% pts** |
| --- | --- | --- |
| **CHARACTERISTIC** | 150  tot |  |
| **Age** |  |  |
| Median | 60 yrs |  |
| Range | 17-82 yrs |  |
| **Germline BRCA mutation status** |  |  |
| Wildtype | 62 | 41% |
| BRCA1 | 16 | 11% |
| BRCA2 | 2 | 1% |
| Unknown | 70 | 47% |
| **2014 FIGO Stage** |  |  |
| II | 5 | 3% |
| III | 125 | 83% |
| IV | 20 | 13% |
| **Surgical outcome** |  |  |
| Complete macroscopic resection | 108 | 72% |
| Incomplete resection* | 3 | 2% |
| Not resectable | 39 | 26% |
| **Grade∞** |  |  |
| Low (grade 1) | 16 | 11% |
| High (grade 2-3) | 134 | 89% |
| **Histology** |  |  |
| High grade serous | 108 | 72% |
| High grade endometrioid | 3 | 2% |
| Clear Cell | 2 | 1% |
| Other high grade® | 21 | 14% |
| Low grade serous | 13 | 9% |
| Mucinous | 3 | 2% |
| **Management** |  |  |
| NACT + surgery | 138 | 92% |
| Primary upfront surgery | 12 | 8% |
| **Chemotherapy type** |  |  |
| Carboplatin and paclitaxel | 145 | 97% |
| Other | 5 | 3% |

Supplementary Table 1 : clinical and pathological characteristics * Known pathological lymph nodes not resected in 3 patients due to age or risk of surgical morbidity. ∞ For serous histology the MD Anderson two-tier grading system was used (Malpica 2004 American Journal of Surgical Pathology – abstract only need to endnote). For other histologies the Silverberg 3-tier (architecture, atypia, mitoses) grading system was used (Silverberg Cancer 1998 reference need to endnote) ®Other high grade histologies includes mixed histology or poorly differentiated NOS.

| **n=53** | pre | post | pre*post (Exact McNemar test) |
| --- | --- | --- | --- |
| IE CD3+/ FOXP3+  ≤1 >1 finite >1 ∞ | 11 32 10 | 4 38 11 | \|  \| post ≤1 \| post >1 \| total \| p-value \| \| --- \| --- \| --- \| --- \| --- \| \| pre ≤1 \| 0 \| 11 \| 11 \| 0.12 \| \| pre >1 \| 4 \| 38 \| 42 (79%) \| \| total \| 4 \| 49 (92%) \| **53** \|  \| |
| stromal CD3+/ FOXP3+  ≤1 >1 finite >1 ∞ | 3 49 1 | 2 51 0 | \|  \| post ≤1 \| post >1 \| total \| p-value \| \| --- \| --- \| --- \| --- \| --- \| \| pre ≤1 \| 0 \| 3 \| 3 \| 1.00 \| \| pre >1 \| 2 \| 48 \| 50 (94%) \| \| total \| 2 \| 51 (96% \| **53** \|  \| |

Supplementary Table 2 : Number and percentage of tumors with evaluable intraepithelial and stromal CD3/FOXP3 ratio pre-NACT and post-NACT.

| **n=53** | pre | post | pre*post (Exact McNemar test) |
| --- | --- | --- | --- |
| IE CD8+/ FOXP3+  ≤1 >1 finite >1 ∞ | 18 25 10 | 12 30 11 | \|  \| post ≤1 \| post >1 \| total \| p-value \| \| --- \| --- \| --- \| --- \| --- \| \| pre ≤1 \| 6 \| 12 \| 18 \| 0.24 \| \| pre >1 \| 6 \| 29 \| 35 (66%) \| \| total \| 12 \| 41 (77%) \| **53** \|  \| |
| stromal CD8+/ FOXP3+  ≤1 >1 finite >1 ∞ | 13 39 1 | 3 50 0 | \|  \| post ≤1 \| post >1 \| total \| p-value \| \| --- \| --- \| --- \| --- \| --- \| \| pre ≤1 \| 1 \| 12 \| 13 \| 0.01 \| \| pre >1 \| 2 \| 38 \| 40 (75%) \| \| total \| 3 \| 50 (94%) \| **53** \|  \| |

Supplementary Table 3 : Number and percentage of tumors with evaluable intraepithelial and stromal CD8/FOXP3 ratio pre-NACT and post-NACT.

| **n=53** | pre | post | pre*post (Exact McNemar test) |
| --- | --- | --- | --- |
| IE CD4+/ FOXP3+  ≤1 >1 finite >1 ∞ | 28 15 10 | 21 21 11 | \|  \| post ≤1 \| post >1 \| total \| p-value \| \| --- \| --- \| --- \| --- \| --- \| \| pre ≤1 \| 8 \| 20 \| 28 \| 0.30 \| \| pre >1 \| 12 \| 13 \| 25 (47%) \| \| total \| 21 \| 32 (60%) \| **53** \|  \| |
| stromal CD4+/ FOXP3+  ≤1 >1 finite >1 ∞ | 28 24 1 | 29 24 0 | \|  \| post ≤1 \| post >1 \| total \| p- alue \| \| --- \| --- \| --- \| --- \| --- \| \| pre ≤1 \| 19 \| 9 \| 28 \| 1.00 \| \| pre >1 \| 10 \| 15 \| 25 (47%) \| \| total \| 29 \| 24 (45%) \| **53** \|  \| |

Supplementary Table 4 : Number and percentage of tumors with evaluable intraepithelial and stromal CD4/FOXP3 ratio pre-NACT and post-NACT.

**A B**


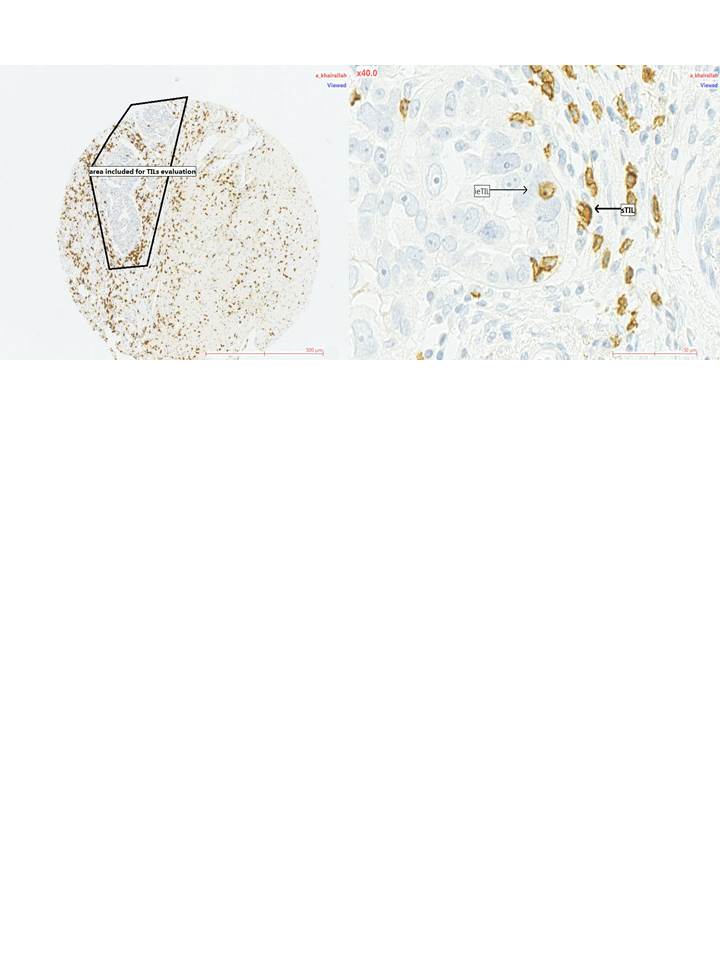


Supplementary Figure 1 : A, identification of the area eligible for assessment of TILs per international TILs Working Group 2014 (x5). B, identification of stromal (sTIL) and intraepithelial (ieTIL) TILs (x40).


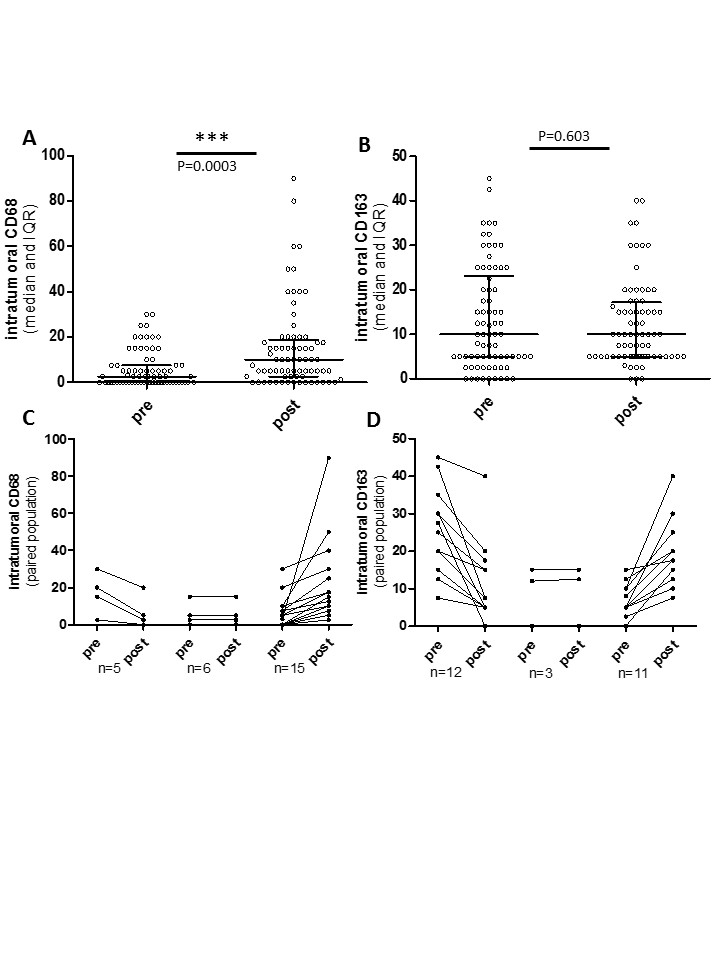


Supplementary Figure 2 : Comparison of TAMs, prior and after neoadjuvant chemotherapy, in all specimens of EOC patients CD68 (A), CD163 (B); and in paired samples, CD68 (C) and CD163 (D).
